# Low-Resolution Molecular Models Reveal the Oligomeric State of the PPAR and the Conformational Organization of Its Domains in Solution

**DOI:** 10.1371/journal.pone.0031852

**Published:** 2012-02-21

**Authors:** Amanda Bernardes, Fernanda A. H. Batista, Mario de Oliveira Neto, Ana Carolina M. Figueira, Paul Webb, Daniel Saidemberg, Mario S. Palma, Igor Polikarpov

**Affiliations:** 1 Institute of Physics of São Carlos, Universidade de São Paulo, São Carlos, São Paulo, Brazil; 2 National Laboratory of Biosciences, CNPEM, Campinas, São Paulo, Brazil; 3 Diabetes Center and Cancer Research Unit, Methodist Hospital, Houston, Texas, United States of America; 4 Department of Biology, Center of Study of Social Insects (CEIS), Institute of Biosciences of Rio Claro, Universidade Estadual de São Paulo (UNESP), Rio Claro, São Paulo, Brazil,; 5 National Institute of Science and Technology on Immunology (INCT/iii), São Paulo, Brazil; University of Hyderabad, India

## Abstract

The peroxisome proliferator-activated receptors (PPARs) regulate genes involved in lipid and carbohydrate metabolism, and are targets of drugs approved for human use. Whereas the crystallographic structure of the complex of full length PPARγ and RXRα is known, structural alterations induced by heterodimer formation and DNA contacts are not well understood. Herein, we report a small-angle X-ray scattering analysis of the oligomeric state of hPPARγ alone and in the presence of retinoid X receptor (RXR). The results reveal that, in contrast with other studied nuclear receptors, which predominantly form dimers in solution, hPPARγ remains in the monomeric form by itself but forms heterodimers with hRXRα. The low-resolution models of hPPARγ/RXRα complexes predict significant changes in opening angle between heterodimerization partners (LBD) and extended and asymmetric shape of the dimer (LBD-DBD) as compared with X-ray structure of the full-length receptor bound to DNA. These differences between our SAXS models and the high-resolution crystallographic structure might suggest that there are different conformations of functional heterodimer complex in solution. Accordingly, hydrogen/deuterium exchange experiments reveal that the heterodimer binding to DNA promotes more compact and less solvent-accessible conformation of the receptor complex.

## Introduction

Peroxisome proliferators activated receptors (PPARs) are members of the nuclear receptor (NR) family, acting as ligand-dependent transcription factors and modulating the activation of cognate genes. There are three different PPAR isotypes: PPARα, PPARβ/δ and PPARγ, which exhibit considerable amino acid sequence conservation. PPARγ plays a central role in the glucose regulation, lipid homeostasis and in the control of the energy balance. Because of this, it has been extensively studied as a molecular target in type II diabetes treatment [1]. PPARγ also stimulates adipose tissue differentiation and functional maintenance [2] and has considerable anti-inflammatory activity [3].

**Figure 1:**
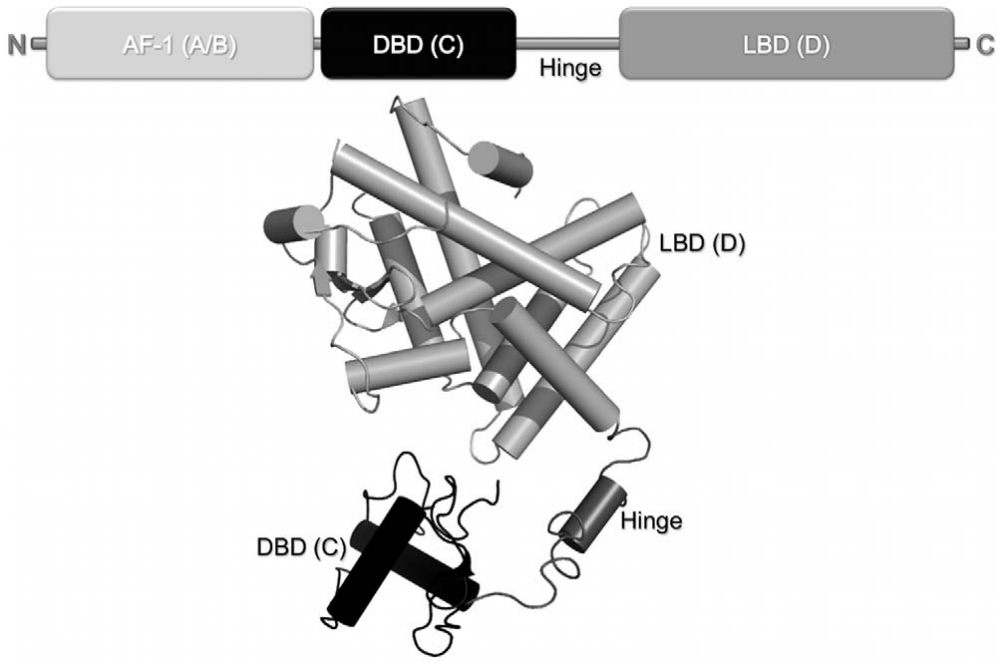
Structural organization of nuclear receptors functional domains. A) Bar representation of nuclear receptors domains. B) Cartoon of crystallographic structure of intact PPARγ+RXRα+DR-1 complex (PDB 3DZU). The N-terminal region (A/B) represented by a light gray bar is absent in the structure because of it high flexibility. The conserved C region, which corresponds to the DBD, is given in black; the LBD, or region E, is shown in gray; and located between C and E domains, the hinge given here in dark gray.

PPARs, like other nuclear receptors, are modular proteins composed of several separable domains [4]. Their N-terminal region (A/B) harbors a ligand-independent activation function 1 (AF-1). The conserved C region corresponds to the DNA binding domain (DBD) and is responsible for sequence-specific DNA recognition. A highly structured E region, or ligand-binding domain (LBD), is responsible for ligand specificity and co-factors recruitment. Hinge or D region is located between C and E domains and is the target of functionally relevant post-translational modifications like phosphorylation and sumoylation [4] (Figure 1).

To understand the function of nuclear receptors at a molecular level, the structural features that mediate heterodimer formation, ligand binding, sequence-specific DNA recognition, and the molecular events underlying the switch from inactive to active receptors must be understood. PPARs activate target-gene transcription upon agonist binding. In this process, PPAR DBDs recognize and bind to specific DNA core motifs known as responsive elements (PPREs), which are direct repeats of two half-sites of the consensus sequence AGGTCA, spaced by one nucleotide. The PPREs are recognized by heterodimers of PPAR with RXR, whereas PPARs alone are unable to bind these DNA response elements [5]. Dimerization is a frequent process in DNA recognition of many eukaryotic transcription factor families [6] and is common within nuclear receptor superfamily, where functional DNA interactions frequently involve homodimers or heterodimers with RXR [7], [8]. PPARγ/RXR heterodimers specifically regulate transcription of genes involved in insulin action, adipocyte differentiation, lipid metabolism and inflammation [9].

The crystallographic structure of intact PPARγ/RXRα heterodimer bound to DNA has recently become available [10]. Overall architectures of the DBD and LBD receptor domains are very similar to the crystallographic structures of the separate domains [11], [12]. However, the full-length structure of nuclear receptor heterodimer bound to DNA PPRE made it possible to study the interactions between functional domains. The two receptors (PPARγ and RXRα) are asymmetrically positioned, with PPARγ and RXRα interactions mediated by well-known interfaces formed by the two LBDs [12] and DBDs. The structures also revealed a third heterodimerization interface between the PPARγ LBD and the DBD and hinge region of RXRα. This interface seems to be modulated by the interactions with DNA, through positioning of both receptors in a unique polarity and spatial arrangement [10].

Although protein crystallography reveals detailed and precise information about tertiary structure of macromolecules, the proteins can adopt other functional conformations. For example, protein conformation is thought to be regulated by DNA contact and chromatin context. The overall shape of a macromolecule and/or its more dynamic quaternary structure in solution can be more reliably accessed by small-angle X-ray scattering (SAXS) [13]. This technique only provides low-resolution structural information relative to X-ray diffraction data, but can reveal overall structure and oligomeric states of native proteins in nearly physiological aqueous conditions, thus permitting analysis of structural changes in response to variations in experimental parameters.

More recently, SAXS and cryo-electron microscopy models of NRs heterodimers revealed alternative conformations for LBD and DBD positions in solution, indicating possible conformational differences in heterodimer arrangements. In addition, the cognate DNA sequences and coactivator presence in the heterodimer seem to result in a more open conformation of the complex. These different conformational states (more closed X-ray structure and more open solution models) might originate from the inherent NRs flexibility [14]. In other words, the NRs crystal structure may reveal only one of the multiple conformational states explored by the receptors.

In order to gather more information about NRs conformations and mobility, here we present systematic analysis of oligomeric state of hPPARγ LBD and LBD-DBD constructs with and without heterodimerization partner hRXRα in the absence of cognate DNA. Furthermore, we also conducted analysis of PPAR solvent accessibility in its monomer and heterodimer forms (with and without DNA) using hydrogen/deuterium exchange (H/D-Ex) monitored by mass spectrometry, which provide additional information about the macromolecular interfaces and the mobility of the complex.

## Results

### Characterization of hPPARγ Monomers and hPPARγ-hRXRα Heterodimers in Solution

**Figure 2:**
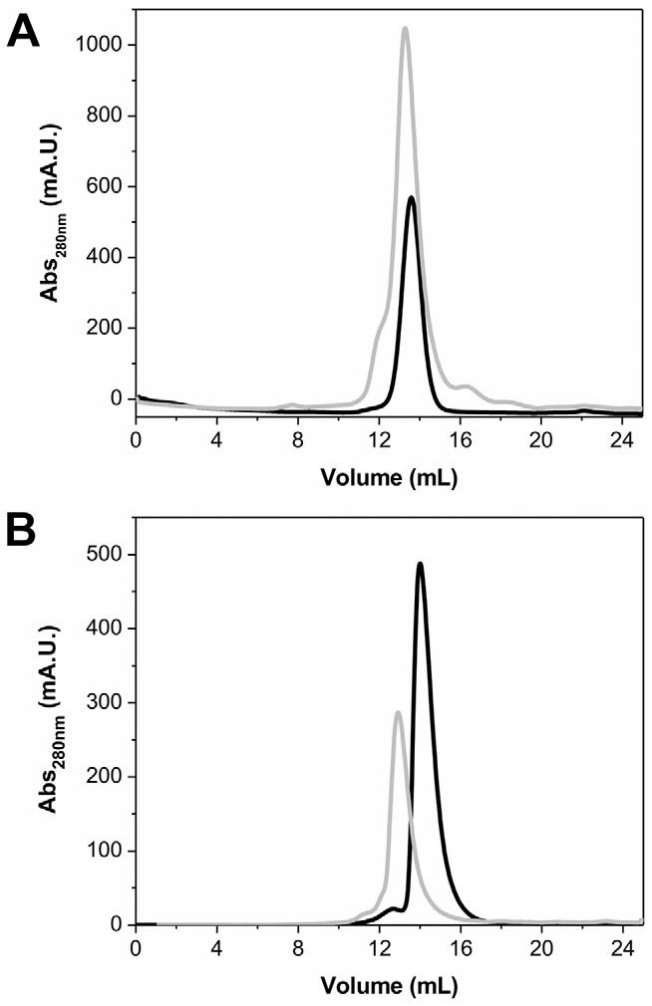
Size exclusion chromatography profile showing the difference in the elution pattern of monomer and heterodimer proteins. A) hPPARγ LBD and hPPARγ/hRXRα LBD and B) hPPARγ DBD-LBD and hPPARγ/hRXRα DBD-LBD. The SEC were performed on a Superdex 75 columm equilibrated with 20 mM Hepes-Na buffer (pH 8.0), 3 mM dithiothreitol, 200 mM NaCl, and 5% glycerol. Monomers are given in black solid lines and heterodimers in gray lines.

We subjected purified preparations of hPPARγ LBD, hRXRα LBD, hPPARγ DBD-LBD and hRXRα DBD-LBD to size exclusion chromatography (SEC). The hPPARγ (LBD and DBD-LBD) showed elution profiles with a single predominant peak (Figures 2A and 2B), corresponding to a hydrodynamic radius (*R_H_*) of 28.6 Å and 35.3 Å, respectively, consistent with hPPARγ LBD and DBD-LBD monomers (apparent molecular weight of approximately 30 kDa and 42 kDa, respectively [15]). After analytical gel filtration, the proteins were submitted to SDS-PAGE (Figure S1), native electrophoresis (Figure S2) and dynamic light scattering experiments (Figure S3) confirming the previous values found to *R_H_* and apparent molecular weight (Table 1). The experimentally determined hPPARγ DBD-LBD *R_H_* value is close to that of thyroid hormone receptor (TR) LBD-DBD monomers [16]. Aiming to examine the influence of the concentration on the *R_H_* values to hPPARγ LBD, the protein, at different concentrations, was submitted to native gel electrophoresis and dynamic light scattering experiments. Both methods of analysis gave the same result, confirming that hPPARγ LBD remains monomeric over a range of protein concentrations from 1 to 20 mg/mL (Figure S2B).

**Table 1:** The hydrodynamic radius (*R_H_*) of the proteins calculated from DLS, Native gel, SEC and SAXS experiments.

Protein	Calculated *R_H_* (Å)			
	*DLS*	*Native Gel*	*Gel Filtration*	*SAXS* †
hPPARγ LBD monomer	27.5	N/C*	28.0	27.4
hPPARγ-hRXRα LBD	39.0	39.0	39.0	36.7
hRXRα LBD dimer	38.0	38.0	36.0	-----------
hRXRα LBD tetramer	43.0	43.0	42.0	-----------

† *R_H_ calculated from R_g_ (Guinier analysis) using the relation between them: R_H_ = R_g_×1.3*.

Since the active form of hPPARγ is a heterodimer with RXR [17], we performed similar studies with the hPPARγ/hRXRα LBD and DBD-LBD heterodimers. The addition of RXR to PPAR (DBD-LBD and LBD) changed the SEC profiles to larger oligomeric forms (Figures 2A and 2B). In this case, complexes were eluted with *R_H_* of 39.0 Å and 47.8 Å, respectively, for LBD and LBD-DBD constructs. The experimentally determined *R_H_* for heterodimer are consistent with values found for hRXRα LBD and NGFI-B LBD dimers, which *R_H_s* are 36.0 Å and 38.5 Å, respectively [18]. The hPPARγ/hRXRα DBD-LBD *R_H_* is also in agreement with *R_H_s* of other NR dimers, such as hTRβ DBD-LBD and hRXRα DBD-LBD that are equal to 42.0 Å [16] and 44.0 Å [19]. Therefore, experimental *R_H_* values indicate that hPPARγ LBDs and hPPARγ DBD-LBDs readily form heterodimers with hRXRα. After analytical gel filtration hPPARγ and hPPARγ/hRXRα were submitted to SDS-PAGE and native electrophoresis to verify complex formation and the stoichiometry of the complexes (Figure S1A and S2).

### Small Angle X-ray Scattering Studies of hPPARγ LBD and Its Heterodimerization with RXR

**Figure 3:**
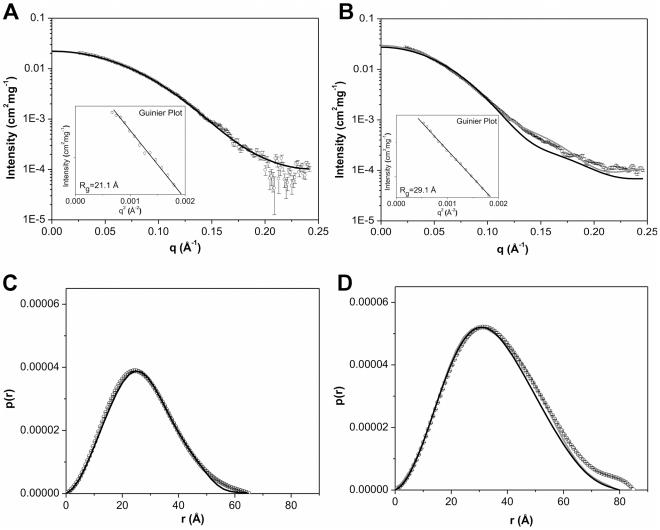
Small-angle X-ray scattering curves for LBD proteins construction. A) hPPARγ LBDs 3.0 mg/mL and B) hPPARγ/hRXRα LBD heterodimers at 3.0 mg/mL. Experimental data (open black circles with errors bars), simulated curves corresponding to the high-resolution model obtained by the use of the PDB id 1FM6 (black solid line) and the rigid body model (gray line). Inset: Guinier plot. The distance distribution function from C) the hPPARγ LBD and D) the hPPARγ/hRXRα LBD. Experimental data (open black circles with errors bars), the high-resolution model (black solid line) and the rigid body model (gray line).

The X-ray scattering curves obtained for hPPARγ LBD and hPPARγ/hRXRα LBD were practically identical at different concentrations, thus indicating the absence of spatial correlation effects over the applied concentration range (Table S1). Therefore, subsequent analysis steps were performed at 3 mg/mL for both hPPARγ LBD and for hPPARγ/hRXRα LBD (Figure 3A and 3B). The Guinier plots gave radius of gyration (*R_g_*) values, which were consistent with monomers hPPARγ LBD and dimers hPPARγ/hRXRα LBD complex (Figures 3A and 3B, inset). Furthermore, these *R_g_* obtained by Guinier analysis showed a good correlation with *R_g_* obtained by the *p(r)* analysis (Figures 3C & 3D and Table 2).

**Table 2:** Structural Parameters Derived from SAXS for hPPARγ LBD (monomer) and hPPARγ/hRXRα LBD (heterodimer).

Parameters	Monomerexp†	Monomerhigh-resolution model ‡	MonomerDAM €	Heterodimerexp†	Heterodimerhigh-resolution model‡	HeterodimerDAM €	HeterodimerRigid body model <$>\raster="rg2"<$>[Table-fn nt105]
**D_max_ (Å)**	65.0±5.0	64.2	62	85.0±5.0	85.4	85.7	89.1
**R_g_ (Å)**	21.0±0.1	20.2	20.1	28.2±0.1	25.5	27.9	26.4
	(Gnom)			(Gnom)			
	21.1	(Crysol)	(Crysol)	29.1	(Crysol)	(Crysol)	(Crysol)
	Guinier			(Guinier)			
**Resolution (Å)**	------------	------------	25.4	------------	------------	25.6	---------------
***MW_SAXS_*** ** (kDa)** §	31.1	------------	------------	65.1	------------	------------	------------
***MW_BSA_*** ** (kDa)** &	30.9	------------	------------	57.9	------------	------------	------------
***MW_SAXS MoW_*** ** (kDa)** #	33.0	------------	------------	73.9	------------	------------	------------
***MW_theoretical_*** ** (kDa)**	31.3	------------	------------	57.8	------------	------------	------------

† *Calculated from the experimental data*.

‡ *Values of hPPARγ LBD monomer and hPPARγ/hRXRα LBD heterodimer from the crystallographic model data (PDB id 1FM6)*.

€ *Parameters of the Dummy Atom Models*.

<$>\raster="rg1"<$>*Parameters of Rigid Body Model. Resolution: 2π/q_max_*.

§ *Experimental estimate of the Molecular Weight (MW) using the forward scattering I(0)/c at the absolute scale using water as a standard* [*21*].

& *MW computed from the scattering data using BSA* [*22*] *as a secondary standard*.

# *Estimate of the MW using SAXS MoW* [*23*].

SAXS data are consistent with the results of SEC, native gel electrophoresis and dynamic light scattering analysis. The obtained structural parameters are also similar to the SAXS studies of NGFI-B LBD dimers (*R_g_* = 28.9 Å and a *D_max_* = 90.0 Å) [20].

The three SAXS-based methodologies used to calculate the molecular weights, which included absolute scattering intensity using water and BSA as standards [21], [22] and SAXS MoW web tool [23], consistently reveal monomers of hPPARγ and heterodimers of hPPARγ/hRXRα in solution (Table 2). It is interesting to note, that the molecular weights predicted by SAXS MoW for the hPPARγ/hRXRα LBD and hPPARγ/hRXRα LBD-DBD heterodimers are somewhat overestimated. Since SAXS MoW algorithm is based on the assumption of the fixed protein density per volume occupied by the molecular envelope [23], this might be a consequence of conformational mobility of the heterodimers (see Discussion sections).

**Figure 4:**
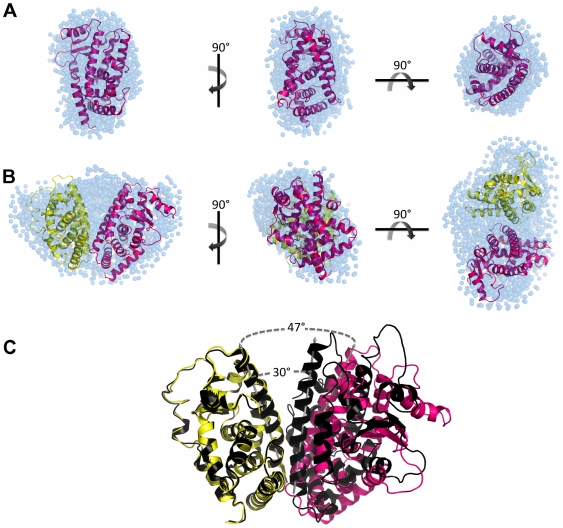
SAXS models for LBD proteins construction. Three orthogonal views of the SAXS *ab initio* models for A) hPPARγ LBD, obtained by Gasbor (shaded spheres), superposed to the hPPARγ LBD monomeric part of the high-resolution model PDB id 1FM6 (cartoon) and B) hPPARγ/hRXRα LBD heterodimer, obtained by Gasbor (shaded spheres), superposed to the rigid body model from PDB id 1FM6 (cartoon). C) Superposition of the rigid body model with the crystallographic structure (PDB id 1FM6) showing the opening angle imposed on the rigid body model being larger than the crystallographic structure. hPPARγ LBD (pink), hRXRα LBD (yellow), crystallographic structure of heterodimer (black) and DAM (blue).

Ten independent *ab initio* simulations were performed with Gasbor package [24] without any symmetry restrictions and of those 252 dummy atoms were attributed to the final model of the monomer and 611 dummy atoms for the heterodimer (Figure 4). The dummy atoms models, PPARγ LBD monomer and hPPARγ/hRXRα LBD heterodimer have a maximum diameter (*D_max_*) of 65.0 Å 85.0 Å, respectively. The models generated for the protein monomer showed globular shape, as expected according to the crystallographic structure for this domain, while the generated heterodimer model showed a more elongated shape, quite different from the former models. Overall, the computed scattering and *p(r)* curve based on the crystallographic structure of monomeric hPPARγ LBD exhibited reasonable fit to the experimental scattering curve (Figure 3 and Table 2). Our low-resolution hPPARγ LBD DAM is also in a good agreement with the crystallographic structure of a single ligand-binding domain of hPPARγ (Table 2 and Figure 4A).

Conversely, SAXS scattering data for hPPARγ/hRXRα LBD complex are not fully compatible with the simulated scattering data computed from the hPPARγ/hRXRα LBD heterodimer crystallographic structure (PDB id 1FM6) [12] (Table 2). Essentially, the heterodimer model needs to be more open than the crystallographic structure to fit experimental SAXS data. Selecting and keeping the fundamental contacts to maintain the known heterodimer interface [12], we performed the rigid body adjustments of the crystallographic model based on our SAXS curves (Table 2). The resulting rigid body model shows a more open heterodimer, with an opening angle between LBDs of about 47 degrees, whereas the opening angle of the crystallographic structure is close to 30 degrees (Figure 4C). This means that the solution dimer interface is likely to be considerably smaller than that observed in the crystal structure (PDB id 1FM6). Numerically, the crystal structure heterodimer interface has an area of 1054.9 Å, while the interface of body rigid model generated has an area of 480 Å, according to “*Protein interfaces, surfaces and assemblies service” (PISA) at European Bioinformatics Institute* (http://www.ebi.ac.uk/pdbe/prot_int/pistart.html) [25]. The rigid body adjustments of the hPPARγ/hRXRα LBD resulted in a considerably better fit to SAXS experimental data (Table 2).

The superposition of the high-resolution structure monomer hPPARγ LBD with the *ab initio* DAM, performed with the program Supcomb, is shown in Figure 4A. The same approach was taken for the superposition of the heterodimer hPPARγ/hRXRα LBD rigid body model with its *ab initio* DAM (Figure 4B). Both models fit known tertiary structural organization well.

### The presence of DBD does not influence hPPARγ oligomeric state

**Figure 5:**
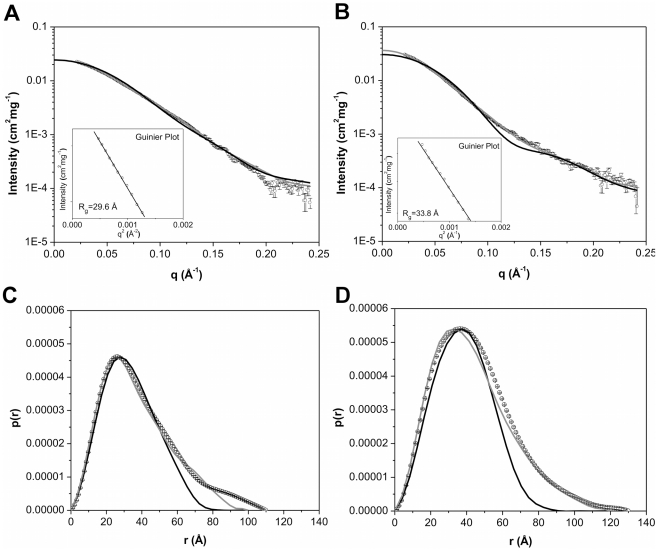
Small-angle X-ray scattering curves for DBD-LBD proteins construction. A) hPPARγ DBD-LBD and B) hPPARγ/hRXRα DBD-LBD, both at 3.0 mg/mL. Experimental data (open black circles with errors bars), simulated curves corresponding to the high-resolution model obtained by the use of the PPARγ monomer from the PDB id 3DZU (black solid line) and the rigid body model (gray line). Inset: Guinier plot. Distance distribution function from C) the hPPARγ DBD-LBD and D) the hPPARγ/hRXRα DBD-LBD. Experimental data (open black circles with errors bars), the high-resolution model (black solid line) and the rigid body model (gray line).

SAXS studies of a PPARγ construct consisting of both DBD and LBD (hPPARγ DBD-LBD) were conducted to study how the DBD influences hPPARγ oligomeric state. The X-ray scattering curves obtained for protein solutions at the different concentrations did not show any spatial correlation effects (Table S1). Typical scattering curves obtained for hPPARγ DBD-LBD monomer and hPPARγ/hRXRα DBD-LBD heterodimer are shown, respectively in Figures 5A and 5B. The structural parameters derived from these curves are given in Table 3. The *R_g_* values are approximately 30.0 Å and 35.0 Å for hPPARγ DBD-LBD monomer and hPPARγ/hRXRα DBD-LBD heterodimer, respectively. These values are compatible with the estimates obtained from the Guinier analysis (Table 3; Figures 5A and 5B, inset), and they are consistent with expected for respective monomers for hPPARγ and heterodimers for their complexes with hRXRα. Moreover, they can be confirmed by the curve obtained on the basis of distances distributions (*p(r)*) (Figure 5C and 5D).

**Table 3:** Structural Parameters Derived from SAXS for hPPARγ DBD-LBD (monomer) and hPPARγ/hRXRα DBD-LBD (heterodimer).

Parameters	Monomerexp†	Monomerhigh-resolution model ‡	MonomerDAM €	MonomerRigid body model ???	Heterodimerexp†	Heterodimerhigh-resolution model ‡	HeterodimerDAM €	HeterodimerRigid body model <$>\raster="rg2"<$>
**D_max_ (Å)**	100.0±5.0	83.1	110.6	101.1	130.0±5.0	92.1	129.1	121.7
**R_g_ (Å)**	31.1±0.1	25.8	31.1	29.5	35.0±0.2	28.2	34.9	34.4
	(Gnom)				(Gnom)			
	29.6	(Crysol)	(Crysol)	(Crysol)	33.8	(Crysol)	(Crysol)	(Crysol)
	(Guinier)				(Guinier)			
**Resolution (Å)**	-----------	------------	26.0	------------	------------	------------	26.0	------------
***MW_SAXS_*** ** (kDa)** §	54.9	------------	------------	------------	77.0	------------	------------	------------
***MW_BSA_*** ** (kDa)** &	54.6	------------	-----------	----------	76.6	------------	------------	------------
***MW_SAXS MoW_*** ** (kDa)** #	55.0	------------	------------	------------	93.9	------------	------------	------------
***MW_theoretical_*** ** (kDa)**	42.0	------------	------------	------------	80.2	------------	------------	------------

† *Calculated from the experimental data*.

‡ *Values of hPPARγ DBD-LBD monomer and hPPARγ/hRXRα DBD-LBD heterodimer from the crystallographic model data (PDB id 3DZU)*.

€ *Parameters of the Dummy Atom Models*.

<$>\raster="rg1"<$>*Parameters of Rigid Body Model. Resolution: 2π/q_max_*.

§ *Experimental estimate of the Molecular Weight (MW) using the forward scattering I(0)/c at the absolute scale using water as a standard* [*21*].

& *Experimental estimate MW using BSA* [*22*] *as a secondary standard*.

# *Estimate of the MW using SAXS MoW* [*23*].

**Figure 6:**
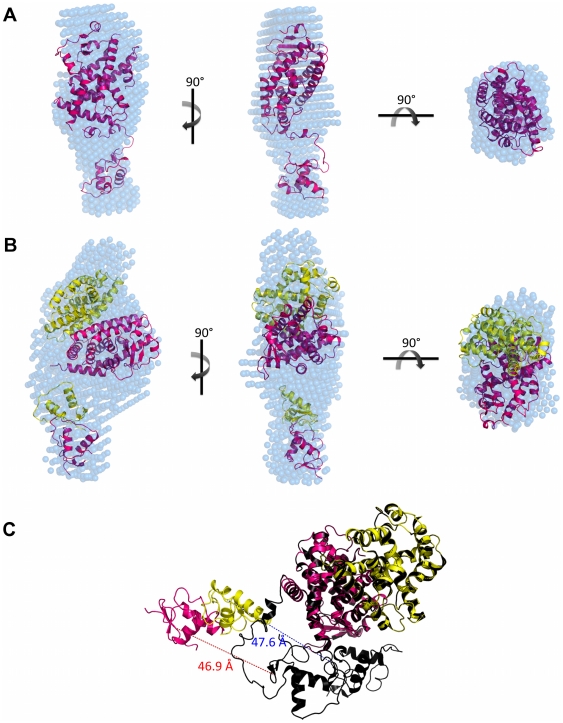
SAXS Models for LBD proteins construction. Three orthogonal views of the SAXS *ab initio* envelope for A) hPPARγ DBD-LBD, obtained by Dammin package, superposed to the monomer rigid body model from PDB id 3DZU and B) hPPARγ/hRXRα DBD-LBD heterodimer, obtained by Dammin package, superposed to the heterodimer rigid body model from PDB id 3DZU. C) Superposition of the rigid body model with the crystallographic structure (PDB id 3DZU) showing the differences between the DBDs positions of the rigid body model and the crystallographic structure. The DBDs was translated and the distance between the initial and final position of them is represented by red dotted line for DBDs hPPARγ and blue dotted line for DBDs of hRXRα. In pink is hPPARγ LBD and yellow is hRXRα LBD of rigid body model; heterodimer crystallographic structure (dark pink and dark yellow) (PDB id 3DZU) and DAM (blue).

The particle shapes (DAMs), computed using Dammin package [26], reveals that one of the molecular envelopes is consistent with monomeric protein (this is the case for hPPARγ DBD-LBD) and another one with the heterodimer (hPPARγ DBD-LBD in the presence of hRXRα DBD-LBD). The molecular DAM for hPPARγ DBD-LBD monomer has a packing radius of about *r_a_* = 2.8 Å, with a maximum diameter *D_max_* = 110.6 Å, whereas molecular envelope for the hPPARγ-hRXRα DBD-LBD heterodimers has a packing radius *r_a_* = 3.3 Å, with a maximum diameter *D_max_* = 129.1 Å, respectively (Figure 6). The experimental SAXS curves and scattering curves computed from the DAMs show good agreement (Table 3). Molecular weights computations using three different methods based on SAXS analysis also confirmed the oligomeric states of hPPARγ DBD-LBD and hPPARγ/hRXRα DBD-LBD as being monomer and dimer, respectively (Table 3).

### Dummy Atom Model Reveals More Open Conformation of hPPARγ/hRXRα in Solution as Compared to High Resolution X-ray Structure of the Complex

DAM generated by Dammin package for hPPARγ/hRXRα DBD-LBD is prolate, elongated and has an asymmetric form. This asymmetry was partly expected based on the arrangement of the domains in the crystallographic heterodimer formed by hPPARγ and hRXRα, which is non-symmetric, allowing several contacts of hPPARγ LBD with other domains of both proteins of the complex with LBD and DBD of hPPARγ closely positioned, and hRXRα LBD and DBD far apart with the space between them filled by the hPPARγ LBD [10].

To compare our low resolution SAXS data with the crystal structure, we computed the theoretical SAXS curves and the pair-distance distribution function for the crystal structures of hPPARγ DBD-LBD monomer and hPPARγ/hRXRα DBD-LBD heterodimer (Figure 5). The crystallographic models do not fit well to the DAMs derived from our SAXS experiments. The profiles of the distance distribution functions *p(r)* corresponding to DAMs and generated for crystallography structures are typical for elongated particles. Nevertheless, the *D_max_* of the DAMs are larger than the crystallographic structure, which indicates that the protein in solution is more elongated than in the crystal.

Rigid body models were generated to minimize discrepancy between crystallographic and experimental models. For the PPAR LBD-DBD monomer rigid body model, the hinge was maintained and the protein domains were separated into two rigid bodies.

Discrepancy between our SAXS data and crystallographic model for the heterodimer could stem from the absence of DNA in our samples and/or from the fact that the SAXS measurements were performed in solution, conditions under which the protein did not have restrictions imposed by the crystalline environment. Thus, the rigid body model generated with the Sasref package [27] was introduced to improve the quality of the fits of experimental SAXS curves to the generated model. This was done by separating their relative domain positions and orientations determined to minimize the differences between the experimental data and the model predictions. The hinge was excluded from computations since the resolution of the SAXS model is not sufficient to define its position and conformations. As mentioned in the Introduction, there was an unexpected intramolecular interface in the crystallographic structure of intact hPPARγ/hRXRα complex [10], which allows interaction of the DBD of hPPARγ with the hinge of hRXRα. In our rigid body model, this interaction could not be observed. This interaction was also not observed in the SAXS experiments performed by another group that studied the envelopes of this complex in the presence of DNA (hPPARγ/hRXRα DBD-LBD+DR-1) [14]. The rigid body model obtained in these studies reflects distant and dissociated positions of DNA and ligand binding domains. This contrasts with the crystal structure [10] which shows a compact conformation of full-length nuclear receptors complex, but it is very consistent with our SAXS measurements, providing envelopes of the same complex but in the absence of DNA.

Our solution SAXS measurements performed with the complex at the absence of DNA reveals that: 1) hPPARγ DBD-LBD forms heterodimers with hRXRα DBD-LBD; 2) the heterodimer is asymmetric; and 3) it has a more extended and elongated shape induced by further separation of hPPARγ/hRXRα LBD and DBD. These structural differences can be observed in the *p(r)* function (Figure 5D), for which the value of *D_max_* for the SAXS model exceeds the value of the crystallographic structure, ensuring a less globular form of the sample in solution. As a result of the rigid body modeling, the two LBDs were positioned in the most bulky part of envelope and the DBDs were positioned in an asymmetric way along the envelope (Figure 6C). This model predicts that the third dimerization interface created by LBD of PPAR and DBD of RXR will not be maintained. Additionally, there are marked differences in the spacing of DBDs and LBDs in the crystallographic structure and the generated rigid body model, which reveals the widely separated domains (Figure 6C). To comply with the SAXS data, the DBD of the PPAR and RXR were translated from initial model (PDB id 3DZU), respectively, on 46.9 Å and 47.6 Å. Our rigid body model describes the small-angle X-ray scattering curves well, and has highly improved the fitting as compared to the X-ray crystallographic structure of the complex (Figure 5 and Table 3).

### Structural Dynamics and Molecular Interfaces of PPAR, PPAR/RXR and PPAR/RXR+DR-1 as Analyzed by Mass Spectrometry

The dynamic behavior and the interface-protected regions of PPAR/RXR heterodimer in solution were analyzed by hydrogen-deuterium exchange experiments analyzed by mass-spectrometry (H/D-Ex MS). In H/D-Ex MS of hPPARγ/hRXRα complex, we identified 51 peptides for hPPARγ, covering 92% of its amino acid sequence (Figure S4A). The deuterium uptake rate was higher for hPPARγ alone, intermediate for the hPPARγ/hRXRα heterodimer and very low for hPPARγ/hRXRα+DNA complex (Figure S4B). Specifically, the uptake rates were 30%, 22% and 10% of D_2_0 incorporation, respectively. The differences in deuterium uptake between the preparations reflect increased compactness and lower flexibility of the more structured complexes with cognate DNA and/or heterodimerization partner, in comparison to the hPPARγ alone. In addition, through measures of different deuterium incubation times, the kinetics of deuterium incorporation seems to be fast, since 15 and 30 minutes of incubation experienced no expressive variation (Figure S4B).

**Figure 7:**
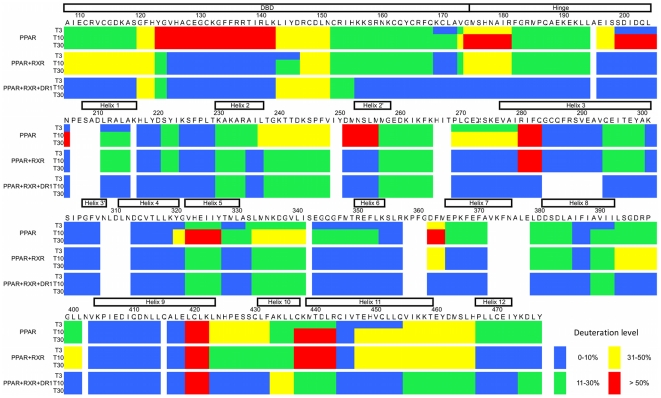
Deuteration level of PPAR monomer and in complex with RXR and DR-1. Deuteration level according with the PPAR sequence, showing the dynamic features of the protein in solution. Each block of three lines represent one protein sample (PPAR monomer, PPAR+RXR complex in absence and in presence of DR-1) in three different deuterium incubation time (3, 10 and 30 minutes). The sequence colored according to H/D-Ex data, considering blue as 0–10%, green 11–30%, yellow 31–50% and red >50% of D_2_O incorporation.

**Figure 8:**
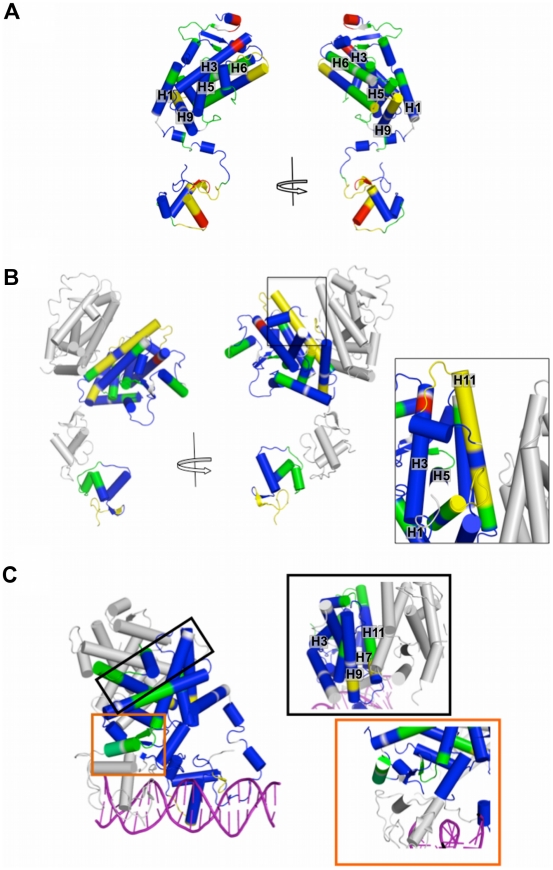
PPAR DBD-LBD models colored according to H/D Ex-data. Protections and solvent exposure are colored according deuteration level, from blue (0–10% D_2_O incorporation), green (11–30% D_2_O incorporation), yellow (31–50% D_2_O incorporation) to red (more that 50% of D_2_0 incorporation). A) hPPARγ monomer; B) hPPARγ/hRXRα heterodimer. The box shows in details the dimerization interface, with H10-11 being not very strongly protected (yellow - 11 to 49% D_2_O incorporation). C) hPPARγ/hRXRα+DR1 complex, the boxes show dimerization interface (top box, framed in black), which presents H10-11 and H7 more protected than that in hPPARγ/hRXRα heterodimer alone; and the third heterodimerization interface (bottom box – orange) indicating higher degree of protection.

The differences in the D_2_O uptake behavior of hPPARγ, hPPARγ/hRXRα and hPPARγ/hRXRα with cognate DNA response element (DR-1) were observed, and as expected, the deuterium incorporation profiles for hPPARγ monomer show that it is more flexible and solvent-expose than the other complexes (Figure 7). The DBD are subject to a high degree of H/D exchange, mainly in the region comprising the first helix (amino acids 123–143). Surprisingly, the hinge domain appears more protected than expected. Perhaps, it might be because of its position close to the receptor's body, as revealed by our SAXS model (Figure 8A). The LBD is by far the most structured and rigid domain, showing low overall H/D exchange (deuterium incorporation below 40%) and the main core (H1, H3, H5, H6 and H9) very well protected.

Overall, the hPPARγ/hRXRα heterodimer is more protected than hPPARγ alone (Figure 8B). The DBD protections show the footprint of DBD dimerization interface (between H9 and H11), which is in accordance with direct repeat array. The hinge is more flexible, disordered or exposed to the solvent, when compared to the hPPARγ alone, suggestive of local protein unfolding, which could be necessary for interaction between the domains of the complexes. The main differences between the hPPARγ/hRXRα heterodimer and hPPARγ monomer are located in the LBD. The LBD core (H1, H3, H5, H6) becomes more structured and compact, with many protected areas. The dimerization interface has a medium level of deuterium incorporation (31% to 50%), H7 is strongly protected and H11 is more accessible, indicating asymmetry of this interface, in compliance with our hPPARγ/hRXRα LBD SAXS model (Figure 7 and 8). Coupled with SAXS analysis which suggests that hRXRα DBD and hPPARγ LBD are far apart and unable to form the interface, this finding represents further evidence that the third interface does not form in solution and in the absence of DNA, and the hPPARγ/hRXRα heterodimer adopts an intermediate state, more compact than that found in the separate proteins, but less packed together than the crystallographic complex (hPPARγ/hRXRα+DR-1 DNA element).

The presence of DNA induces an even more solvent protected conformation in the heterodimer hPPARγ/hRXRα (Figure 8C), which is more consistent with the crystallographic structure (PDB id 3DZU). The DBDs and hinges of both subunits of the heterodimers become more protected. Further example is the hinge region (residues 154–195), which display lower mobility, presumably because of its possible interactions with DNA.

The LBD protein core is also significantly more protected from solvent. Significantly lower dynamic exchange as compared to other samples (less than 10% of D_2_O incorporation) was observed for the H9 and H11 (mainly responsible for dimerization interface). This might indicate that the interface becomes larger and more symmetric. Consequently, the hPPARγ/hRXRα heterodimer bound to DNA seems to be more compact and further stabilized by the DNA addition.

In addition to the protected regions belonging to the domains core, the region comprising loops formed by residues 110–120 of DBD and the 378–385 and 422–431 of LBD showed higher protection to H/D exchange. Analyses of the crystallographic structure the hPPARγ/hRXRα complex reveals that these parts of the structure become more internalized in the presence of DNA. This does not happen in the absence of DNA, because of the extended conformation of the heterodimer. These observations are in agreement with the hypothesis that the presence of the DNA will trigger the rearrangements of the hPPARγ/hRXRα dimer conformation toward a more compact state.

The third heterodimerization interface also displays stronger protection as compared to a complex without DNA, as can be seen for H7, for example, as well as some parts of the LBD surrounding this interface (H6 and H3). Together, these findings suggest that there is an increase in overall compactness and reorganization in the protein complex when the DNA is added. Furthermore, the dimerization interfaces of hPPARγ/hRXRα heterodimer in solution, is different from the interfaces of hPPARγ/hRXRα+DR-1 complex in the crystalline state.

## Discussion

PPARγ has a central role in the regulation of glucose and lipid homeostasis and is involved in inflammatory processes and is an important drug target for treatment of Type 2 Diabetes and inflammation [28], [29]. While the crystallographic structure of the complex of full length PPARγ and RXRα is known, structural alterations induced by heterodimer formation and DNA contacts in solution are not well understood. In order to expand knowledge about the molecular shape, oligomeric state and protein-protein interaction of hPPARγ in solution alone and in the presence of its heterodimerization partner, hRXRα, we performed SAXS analysis and H/D exchange studies.

Although PPARs associate with RXR in the presence of ligand in living cells [30]–[32], its oligomeric state in solution had not been explored. Our SAXS-derived structural parameters, supported by SEC, native electrophoresis and DLS are consistent with the monomeric form of both, hPPARγ LBD and DBD-LBD, constructs in solution, even at high protein concentrations required for SAXS experiments. This is highly unusual since other nuclear receptors, studied to date in solution by SAXS and other techniques, form dimers and higher oligomeric forms [18]–[20], [33]. Nevertheless, our SAXS experiments reveal that in the presence of hRXRα, both hPPARγ LBD and DBD-LBD protein constructs readily form heterodimers. This suggests that our hPPARγ preparations are comprised of functional protein, which retains the capacity to heterodimerize with RXR and to bind to DNA, essential steps in eliciting its functional activity, and confirms that hPPARγ is a constitutive monomer with a high capacity for heterodimerization.

Fitting of high-resolution X-ray structural models into our low-resolution SAXS models revealed unexpected differences between organization of the heterodimer in the crystal and in solution. The SAXS-based rigid body model constructed for LBDs render the hPPARγ/hRXRα heterodimer considerably about 17 degree more open relative to high-resolution hPPARγ/hRXRα LBD crystallographic structure [12]. In addition, our SAXS experiments performed on hPPARγ/hRXRα DBD-LBD complex reveals that this heterodimer becomes asymmetric and adopts a more extended and elongated shape as compared to the conformation found in the crystal structure [10], which are in agreement with SAXS envelopes of these proteins in complex with DNA [14]. This elongated form of the hPPARγ/hRXRα DBD-LBD heterodimer in solution is induced by further separation of hPPARγ LBD and DBD with respect to one another.

Our H/D-Ex experiments also revealed differences in hPPARγ, hPPARγ/hRXRα and hPPARγ/hRXRα+DR-1 species in terms of solvent accessibility. Our results indicate that hPPARγ/hRXRα heterodimer alone, in the absence of DNA, is an intermediately condensed form, which is stabilized by the cognate DNA binding. Essentially, the asymmetric dimerization interface between hPPARγ/hRXRα LBDs, became more protected after DR1 binding. Finally, our data predicts that the third dimerization interface, between DBD of hRXRα and LBD of hPPARγ, could be formed only in the presence of DR1 DNA, as predicted from analysis hPPARγ/hRXRα+DR1 tridimensional structure [10], but the open and close conformation of the complex remain in a dynamic equilibrium.

**Figure 9:**
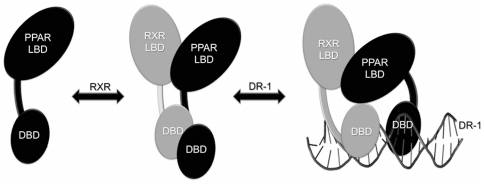
Cartoon schematically representing the mechanism of heterodimerization and binding to the DNA. When the PPAR is activated, it recruits RXR, forming an intermediary heterodimer, which has the LBDs and DBDs domains in extended and open conformation. Following to DNA binding, the PPAR/RXR heterodimers suffer additional conformational changes, becoming more condensed and less solvent-exposed.

Our results shed more light on the functionally relevant heterodimer hPPARγ/hRXRα formation and the hPPARγ behavior in solution. Based on our studies, we purpose a following model of PPAR activation (Figure 9). According to this model, ligand-bound PPAR recruits RXR and forms an intermediary heterodimer, more stable then the PPAR alone, but with LBD heterodimer surfaces relatively open as compared to the crystallographic model. The DBDs show extended conformations, separated from the LBDs, as revealed by our SAXS model. After DNA binding, this intermediary heterodimer undergoes additional conformational changes, caused by the interactions between the receptors and DNA, and becomes more compact, able to adopt the conformation similar to the one revealed by the crystallographic structure [10]. Thus, our data confirms that DNA could induce significant changes in the interactions of DBDs, LBDs and hinge organization of the PPARγ/RXRα complex, consistent with predictions that DNA acts as an allosteric ligand, inducing widespread reorganizations in receptor conformation [34], [35]. Furthermore, the mass experiments showed changes in the deuterium incorporation pattern, after the DNA addition. It will be interesting to understand how these structural alterations may affect PPARγ function on DNA elements versus their actions at alternate elements where direct DNA interaction is not required [36].

## Materials and Methods

### Materials

The bacterial expression vector pET28a(+) was purchased from NOVAGEN. Isopropyl-β-d-thiogalactopyranoside (IPTG) was obtained from Invitrogen, Inc. Talon Superflow Metal Affinity Resin was from BD Biosciences Clontech. Phenylmethylsulfonyl fluoride (PMSF), lysozyme, protein standards used as sodium dodecyl sulfate–polyacrylamide gel electrophoresis (SDS–PAGE) markers and D_2_O (Deuterium oxide) were purchased from Sigma Aldrich. Bradford dye was from Bio-Rad. HiLoad Superdex 75 26/60, HiLoad Superdex 200 16/60 and Superdex 75 HR 10/30 gel filtration columns were purchased from GE Healthcare. All other chemicals were of analytical grade.

### Expression and purification

The human PPARγ LBD (amino acids 204–477), RXRα LBD (amino acids 225–462), PPARγ DBD-LBD (amino acids 101–468), and RXRα DBD-LBD (amino acids 135–462) were inserted into the pET28a(+) (Novagen) and expressed in the *Escherichia coli* strain BL21(DE3).

The same protocol of protein expression and purification was used for all protein studied in this work. Protein expressions were conducted in LB culture and were induced with 1 mM IPTG (isopropyl β-D-1-thiogalactopyranoside), under incubation at 20°C for 3 h. 5 µM of zinc sulfate was added to the culture during expression of the constructs with DBD domains. Cells were collected by centrifugation and the pellets were resuspended in 50 mM sodium phosphate, pH 7.5, 300 mM NaCl, 10% glycerol, 2 mM 2-mercaptoethanol, and 10 mM imidazole (buffer A). Phenylmethylsulfonyl fluoride (PMSF) and lysozyme were present at 10 mM and 250 µg/mL, respectively. The lysate was sonicated, clarified by centrifugation and the supernatant loaded onto a Talon Superflow Metal Affinity Resin (BD Biosciences Clontech, Palo Alto, CA) pre-equilibrated in Buffer A. The bound hPPARγ was eluted with 50 mM sodium phosphate, pH 8.0, 300 mM NaCl, 10% glycerol, 2 mM β-mercaptoethanol, and 300 mM imidazole (buffer B), in a single step. The eluted pool was collected, and the His-tag was subsequently removed (except to hRXRα) by incubation with thrombin at 10 U/mg for 12 h at 18°C. After, as an additional purification step, hPPARγ LBD was loaded into the gel filtration HL Superdex 75 26/60 column and hRXRα LBD and DBD-LBD, and hPPARγ DBD-LBD, into HL Superdex 200 16/60 column (GE Healthcare) equilibrated with 20 mM Hepes-Na buffer (pH 8.0), 3 mM dithiothreitol, 200 mM NaCl, and 5% glycerol.

Protein content and purity were confirmed by coomassie blue-stained sodium dodecyl sulfate polyacrylamide gel electrophoresis (SDS-PAGE). Protein concentrations were determined using the Bradford dye assay (Bio-Rad, Hercules, CA).

### Heterodimer Preparation

The purified protein pairs: hPPARγ DBD-LBD and hRXRα DBD-LBD, or hPPARγ LBD and hRXRα LBD at a concentration of 20 mg/mL each, were incubated in a molar proportion of 1∶1 for 1 h at 4°C. After, each complex was purified by loading onto a Superdex 75 HR 10/30 (GE Healthcare) for LBD constructions, and Superdex 200 HR 10/30 (GE Healthcare) for DBD-LBD constructs. The size exclusion chromatography (SEC) was also used to evaluate the oligomeric species present in solution (Text S1). The column was standardized with the gel filtration calibration kit (GE Healthcare), thyroglobulin, ferritin, catalase, aldolase, albumin, ovoalbumin, chymotrypsinogen, and ribonuclease A (hydrodynamic radii (*R_H_*) of 85.0, 61.0, 52.2, 48.1, 35.5, 30.5, 20.9, and 16.4 Å, respectively), utilized as calibration standards. The elution volumes of these proteins were used to calculate the *K_av_* values according to columns calibration as described [16]. All the eluted samples were checked by SDS-PAGE 15%. Other methodologies were applied to assist in the oligomeric states evaluation (Text S2 and S3).

### Small-Angle X-ray Scattering

#### SAXS experiments

SAXS data for hPPARγ LBD and hPPARγ/hRXRα LBD complex at 1, 3 and 6 mg/mL, as well as, hPPARγ DBD-LBD and hPPARγ/hRXRα DBD-LBD at 1, 3 and 6 mg/mL, were performed at the D02A-SAXS2 beamline of the Synchrotron Light National Laboratory (Campinas, Brazil) (Text S4). Measurements were done with a monochromatic X-ray beam with a wavelength of *λ* = 1.488 Å and the X-ray patterns were recorded using a two-dimensional CCD detector (MarResearch, USA). The sample-to-detector distance was set at 955.3 mm, resulting in a scattering vector range of 0.015 to 0.35 Å^−1^, where *q* is the magnitude of the *q*-vector defined by *q* = 4πsinθ/λ (2θ is the scattering angle). The samples diluted in a gel filtration buffer were centrifuged at 23,500 g for 30 minutes, at 4°C to remove any aggregates or particles and then placed on ice. For SAXS measurements, protein samples were introduced into a 1 mm path length cell with mica windows at 20°C. Two successive frames of 300 s each were recorded for each sample to monitor radiation damage and beam stability. Buffer scattering was recorded before the sample scattering. The SAXS patterns were individually corrected for the detector response and scaled by the incident beam intensity and the sample absorption. The buffer scattering (parasitic scattering from windows, narrows, etc.) was subtracted from the corresponding sample scattering. The integration of SAXS patterns were performed using Fit2D software [37], and the curves were scaled by protein concentration.

#### SAXS data analysis

The radius of gyration, *R_g_* is a global measure of the size and shape of the molecular complex which is related to hydrodynamic radius (*R_H_*) by *R_H_* = *R_g_*×1.3 [38] and was approximated using two independent procedures, by Guinier equation [39] and by indirect Fourier transform method using Gnom package [40]. The distance distribution functions *p(r)* also was evaluated by Gnom and the maximum diameter, *D_max_* was obtained. Molecular weights (MW) were estimated by three methods: (1) by determining the absolute scattering intensity using water scattering (primary standard) (Text S5) [21], (2) by comparison of the forward-scattered intensity with the secondary protein standard, bovine serum albumin (BSA) (Text S6) [22] and (3) using a novel procedure implemented as a web tool SAXS MoW (www.ifsc.usp.br/~saxs/saxsmow.html) [23]. The later procedure does not require the measurement of SAXS intensity on an absolute scale and does not involve a comparison with another SAXS curve determined from a known standard protein. To calculate the forward scattering I(0) in the absolute scale, the known scattering of water equal to 1.632×10^−2^ cm^−1^ at 288 K was used [21].

#### SAXS ab initio modeling

Dummy atom models (DAMs) were calculated from the experimental SAXS data using *ab initio* procedure implemented in either Dammin [26] and Gasbor packages [24]. Several runs of *ab initio* shape determination with different starting conditions led to consistent results as judged by the structural similarity of the output models, yielding nearly identical scattering patterns and fitting statistics in a stable and self-consistent process. Crysol package was used to generate the simulated scattering curves from DAMs [40]. The evaluation of *R_g_* and *D_max_*, were performed with the same package.

#### Fitting of DAMs with crystallographic structures

The crystallographic structures of hPPARγ LBD monomer (PPAR monomer part from the PDB id 1FM6) [12], hPPARγ/hRXRα LBD complex (PDB id 1FM6), hPPARγ DBD-LBD monomer (PPAR monomer part from the PDB id 3DZU) and hPPARγ/hRXRα DBD-LBD complex (PDB id 3DZU) [10] were used to generate the simulated scattering curves by Crysol package [40] and to determine the *R_g_* and *D_max_*. Some of the simulated curves based on the crystallographic structures had good agreement with the experimental SAXS data. The correspondent three-dimensional structures were superimposed with *ab initio* DAMs using the Supcomb package [24]. Figures of the superpositions were generated by the program PyMOL [41].

#### Rigid body modeling

Rigid body modeling was performed for the hPPARγ/hRXRα LBD complex using Sasref package [27]. The two monomers from the crystallographic structure (PDB id 1FM6) were separated and their relative position and orientation were minimized. Based on the known classic dimerization interface between hPPARγ LBD and hRXRα LBD, the intermolecular contacts RXR F415-A433 PPAR and RXR L420–L436 PPAR [12] were maintained during the minimization procedure. In order to improve the quality of fits, the protein domains were allowed to separate and thus their relative positions and orientations were determined by rigid body modeling. For the PPAR LBD-DBD monomer rigid body model, the hinge was maintained and the protein was separated into two rigid bodies maintaining the primary sequence of amino acid residues P206-E207. The rigid body refinement allowed better adjustment of the structure inside the DAM. To perform rigid body modeling with the heterodimer DBD-LBD, we separated the complex into two rigid bodies, one containing the LBDs and the other with the DBDs, since limited structural information of SAXS data did not allowed us to use too many independent domains and more degrees of freedom. The hinge domains (for PPAR, a fragment between A172-P206 and for RXR, the fragment between E203-N227) have been excised from the structural templates. The dimerization interface of LBD was maintained, as it had been described previously for structure with separate LDB domains [12] and also observed in the structure of the full-length receptor [10]. For position of DBDs, we used the dimerization interface described for the DBDs of the estrogen receptor (ER) (PDB 1HCQ), which shows a complementarity of shape as well as a number of direct contacts between domains [42]. Crysol package was used to generate the simulated scattering curves.

### Mass-spectrometry of hPPARγ-hRXRα DBD-LBD

Hydrogen/deuterium exchange coupled with chromatography-mass spectrometry analysis has been extensively used in analysis of proteins and their interactions, including protein∶protein or protein∶ligands interactions and protein dynamics [43]–[47].

Mass-spectrometry experiments were conducted using hPPARγ DBD-LBD alone and the heterodimer hPPARγ/hRXRα DBD-LBD complex in the presence and absence of DNA PPRE (5′-AGCTAAAGGTCAGAGGTCAGTAGGA-3′).

The H/D exchange mass spectrometry experiments started by diluting hPPARγ/hRXRα complexes at high concentration (26 mg/mL) 10 times in D_2_O buffer (final buffer: 2 mM Hepes, 15 mM NaCl, 0.5% Glycerol, 0.3 mM DTT and 60% v/v D_2_O). These mixtures (100 µL) were incubated for 3, 10 and 30 minutes at a room temperature, in order to have mild conditions of hydrogens exchange by deuteriums at the protein surfaces. The kinetics of deuterium incorporation is fast and after 30 minutes of incubation, the H/D exchange becomes stabilized (Text S7). After incubation, the proteins were immediately submitted to pepsin cleavage at a ratio of 1∶50 enzime to protein by mass, for 10 minutes, with addition of 60 µL of 100 mM of sodium phosphate buffer pH 2.5, on ice to avoid H/D back-exchange. After addition of 30% acetonitrile, the samples contained the peptic fragments was immediately applied, to avoid back-exchange with solvent hydrogen, by direct injection onto a Quattro II triple-quadrupole mass spectrometer (Micromass, UK), equipped with a standard ESI source. By the analysis of the displacement in peptide peaks, the fragments of the protein undergoing H/D exchange were identified. The software MS-Digest (The Regents of the University of California) was used to identify the sequence of the peptic peptide ions, generated by pepsin cleavage. The deuterium incorporation level for each peptide was determined from differences in mass centroids between the deuterated and non-deuterated fragments using Masslinx software (Micromass, UK).

## Supporting Information

Figure S1 **SDS-Page of protein purification.** A) hPPARγ LBD, hRXRα LBD and hPPARγ/hRXRα LBD heterodimer purification 15% SDS-Page. Lane 1 molecular weight markers (66, 45, 36, 29 e 12 kDa); Lane 2 hPPARγ LBD elution after the affinity column; Lane 3 hRXRα LBD elution after the gel filtration column; Lane 4 hPPARγ/hRXRα LBD heterodimer elution after the gel filtration column B) hPPARγ DBD-LBD, and hPPARγ/hRXRα DBD-LBD heterodimer purification 15% SDS-Page. Lane 1 molecular weight markers (66, 45, 29 e 12 kDa); Lane 2 hPPARγ DBD-LBD elution after the affinity column; Lane 3 hPPARγ/hRXRα DBD-LBD heterodimer elution after the gel filtration column.
(TIFF)

Figure S2 **Native gel electrophoresis.** A) Lanes 1 and 4 molecular weight markers (440, 232, 140, 66 kDa); Lane 2 hPPARγ LBD; Lane 3 hRXRα LBD; Lane 5 hPPARγ/hRXRα LBD heterodimer. B) hPPARγ LBD in several concentrations. Lane 1 molecular weight markers (440, 232, 140, 66 kDa); Lane 2 hPPARγ LBD 1 mg/mL; Lane 2 hPPARγ LBD 3 mg/mL; Lane 3 hPPARγ LBD 5 mg/mL; Lane 4 hPPARγ LBD 7 mg/mL; Lane 5 hPPARγ LBD 10 mg/mL; Lane 6 hPPARγ LBD 15 mg/mL; Lane 7 hPPARγ LBD 20 mg/mL.
(TIFF)

Figure S3 **Dynamic light scattering results.** The R_H_ of hPPARγ LBD, derived from DLS studies, are plotted as a function of the protein concentration.
(TIFF)

Figure S4 **Analysis of H/D Exchange experiments.** A) PPAR sequence indicating the peptides identified in the H/D Ex experiments. B) D2O uptake by PPAR, PPAR-RXR heterodimer and by PPAR/RXR+DR1 complex, in 3, 10 and 30 minutes of D_2_O incubation. The PPAR monomer is more solvent accessible than the complexes.
(TIFF)

Table S1 **R_g_ values resultant from Guinier analysis for proteins at different concentrations.**
(DOCX)

Text S1 **Size Exclusion Chromatography (SEC).** It was used to evaluate the oligomeric species present in solution.
(DOCX)

Text S2 **Native polyacrylamide gel electrophoresis.** The mobility of individual bands was used to calculate the *R*_H_ of the oligomeric states of proteins.
(DOCX)

Text S3 **Dynamic light scattering.** The protein was submitted to this measure at different concentrations.
(DOCX)

Text S4 **Details of SAXS Experiments.**
(DOCX)

Text S5 **Absolute scale SAXS measurements.**
(DOCX)

Text S6 **The proteins molecular weight determination by SAXS measurements using BSA as a reference.**
(DOCX)

Text S7 **Hydrogen-deuterium exchange experiments analyzed by mass-spectrometry.**
(DOCX)
