# Supplementary figures and images for: Low-Resolution Molecular Models Reveal the Oligomeric State of the PPAR and the Conformational Organization of Its Domains in Solution
Source: PLoS One. 2012 Feb 21;7(2):e31852. doi: 10.1371/journal.pone.0031852 (PMC3283691; doi:10.1371/journal.pone.0031852)

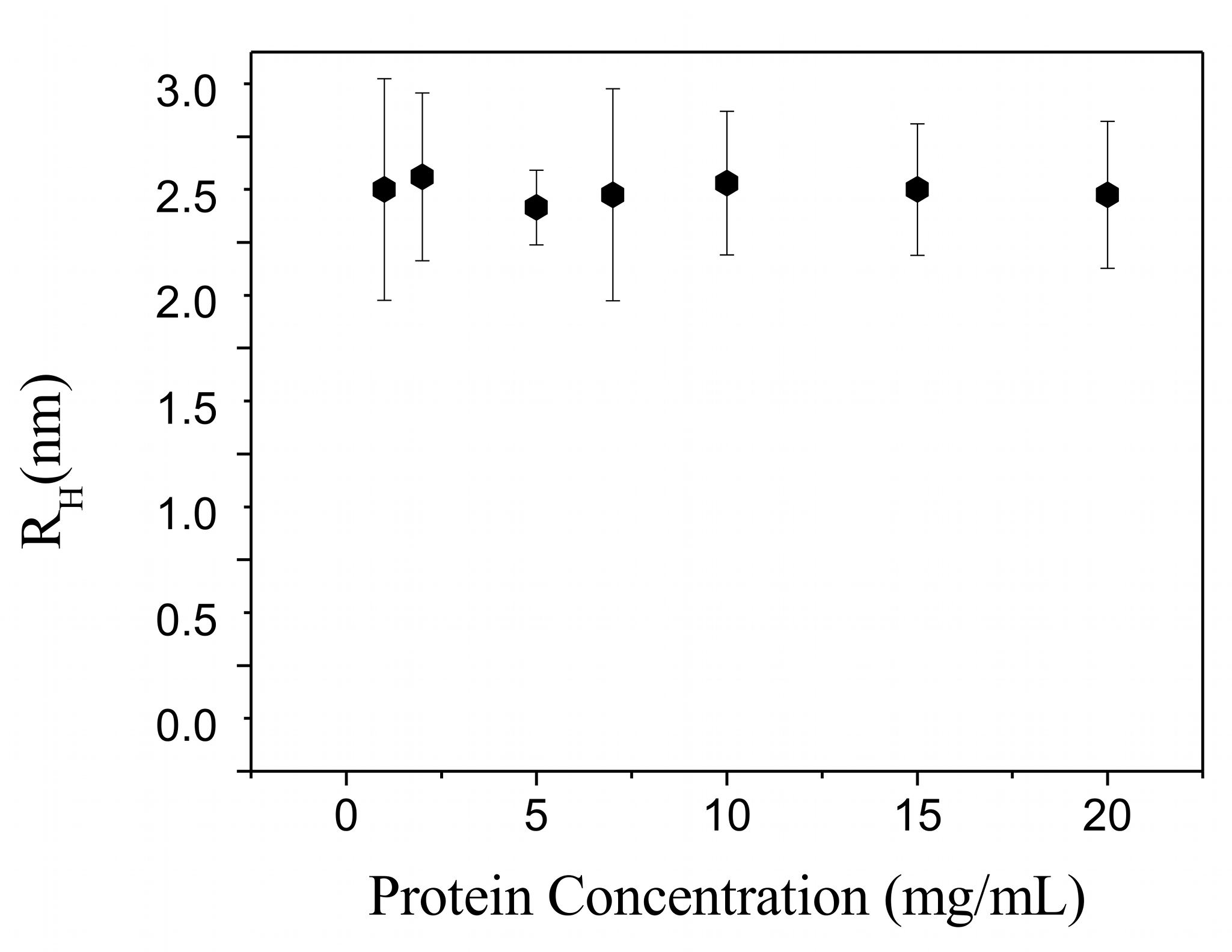

Supplement: Figure S3 — Dynamic light scattering results. The RH of hPPARγ LBD, derived from DLS studies, are plotted as a function of the protein concentration. (TIFF) [file pone.0031852.s003.tif]
