# Supplementary material for: Low-Resolution Molecular Models Reveal the Oligomeric State of the PPAR and the Conformational Organization of Its Domains in Solution
Source: PLoS One. 2012 Feb 21;7(2):e31852. doi: 10.1371/journal.pone.0031852 (PMC3283691; doi:10.1371/journal.pone.0031852)
Supplement: Table S1 — Rg values resultant from Guinier analysis for proteins at different concentrations. (DOCX) [file pone.0031852.s005.docx]

**Table S1:**

|  | **R_g_ (Å) - Guinier analyses** | | |
| --- | --- | --- | --- |
| **Amostra** | **1mg/mL** | **3 mg/mL** | **6 mg/mL** |
| Monomer LBD | 22.6 | 21.1 | 22.4 |
| Heterodimer LBD | 27.8 | 29.1 | 28.2 |
| Monomer DBD-LBD | 30.7 | 29.6 | 30.9 |
| Heterodimer DBD-LBD | 31.4 | 33.8 | 33.9 |
