# Supplementary material for: Low-Resolution Molecular Models Reveal the Oligomeric State of the PPAR and the Conformational Organization of Its Domains in Solution
Source: PLoS One. 2012 Feb 21;7(2):e31852. doi: 10.1371/journal.pone.0031852 (PMC3283691; doi:10.1371/journal.pone.0031852)
Supplement: Text S1 — Size Exclusion Chromatography (SEC). It was used to evaluate the oligomeric species present in solution. (DOCX) [file pone.0031852.s006.docx]

***SUPPORTING INFORMATION***

**Text S1:**

***Size Exclusion Chromatography -*** The size exclusion chromatography (SEC) was applied as a second purification step of proteins samples and it was also used to evaluate the oligomeric species present in solution. The proteins hPPARγ LBD, hPPARγ DBD-LBD and the heterodimers hPPARγ/hRXRα LBD, hPPARγ/hRXRα DBD-LBD were applied onto a Superdex 75 HR 10/30 size exclusion column, which was previously equilibrated with buffer 20 mM Hepes, pH 8.0, 300 mM NaCl, 3 mM DTT, 5% glycerol. The column was standardized with the gel filtration calibration kit (GE Healthcare), thyroglobulin, ferritin, catalase, aldolase, albumin, ovoalbumin, chymotrypsinogen, and ribonuclease A (hydrodynamic radii (*R_H_*) of 8.5, 6.1, 5.22, 4.81, 3.55, 3.05, 2.09, and 1.64 nm, respectively) used as calibration standards. The elution volumes of these proteins were used to calculate the *K*_av_ values [1]. All the samples eluted were check by SDS-PAGE 15% (Figure S1A and S1B)
