# Supplementary material for: Low-Resolution Molecular Models Reveal the Oligomeric State of the PPAR and the Conformational Organization of Its Domains in Solution
Source: PLoS One. 2012 Feb 21;7(2):e31852. doi: 10.1371/journal.pone.0031852 (PMC3283691; doi:10.1371/journal.pone.0031852)
Supplement: Text S2 — Native polyacrylamide gel electrophoresis. The mobility of individual bands was used to calculate the R H of the oligomeric states of proteins. (DOCX) [file pone.0031852.s007.docx]

***SUPPORTING INFORMATION***

**Text S2:**

***Native polyacrylamide gel electrophoresis -*** The samples relative of hPPARγ LBD, hRXRα LBD and hPPARγ/hRXRα LBD heterodimer, at a concentration of 2 mg/mL was electrophoresed onto a 8 to 25% (w/v) gradient polyacrylamide nondenaturing gel (Figure S2A), using the PhastSystem (GE Healthcare), at 4°C, and Coomassie-stained following standard protocols. Another experiment, using this same technique, was done applying different concentrations of hPPARγ LBD (1, 3, 5, 7, 10, 15 and 20 mg/mL) onto a native gel, in order to evaluate the presence of other oligomeric states of this protein (Figure S2B).

Protein standards of known hydrodynamic radii (thyroglobulin, 85 Å; ferritin, 61 Å; catalase, 52.2 Å; lactate dehydrogenase, 43 Å; and bovine serum albumin, 35.5 Å) were electrophoresed in the same conditions. The mobility of individual bands of these standard proteins was plotted as the retardation factor (*R_f_*) versus the hydrodynamic radii (*R*_H_) [1]. The linear equation obtained from this calibration was used to calculate the *R*_H_ of the oligomeric states of hPPARγ LBD, hRXRα LBD, and the heterodimer hPPARγ/hRXRαLBD.
