# Supplementary material for: Low-Resolution Molecular Models Reveal the Oligomeric State of the PPAR and the Conformational Organization of Its Domains in Solution
Source: PLoS One. 2012 Feb 21;7(2):e31852. doi: 10.1371/journal.pone.0031852 (PMC3283691; doi:10.1371/journal.pone.0031852)
Supplement: Text S3 — Dynamic light scattering. The protein was submitted to this measure at different concentrations. (DOCX) [file pone.0031852.s008.docx]

***SUPPORTING INFORMATION***

**Text S3:**

***Dynamic light scattering -*** Dynamic light scattering (DLS) was used to determine the hydrodynamic radius of hPPARγ LBD. The protein was submitted to this measure at different concentrations. Beyond the *R_H_*, this experiment was also done to evaluate sample monodispersity (Figure S3), as described previously [1]. The measurements were carried out in buffer of 20 mM Hepes buffer (pH 8.0), 200 mM NaCl, 1 mM DTT, and 5 % glycerol, using a DynaPro MSTC014 instrument (Protein Solutions Inc., Lakewood, NJ). Thirty acquisitions were averaged in a single measurement with an acquisition time of 2.5 s each, using a 12 µL cuvette, at 4 °C. Five sequential measurements were performed for each concentration of the samples. Hydrodynamic radii (*R_H_*) were transformed to radii of gyration (*R*_G_) by the simplified relationship: *R*_H_ = *R*_G_ x 1.3 [2].

The same approach was used to determine the hydrodynamic radius of hPPARγ/hRXRα LBD complex but instead of several concentrations, the measurements were performed with proteins in a concentration of 1 mg/mL.
