# Supplementary material for: Low-Resolution Molecular Models Reveal the Oligomeric State of the PPAR and the Conformational Organization of Its Domains in Solution
Source: PLoS One. 2012 Feb 21;7(2):e31852. doi: 10.1371/journal.pone.0031852 (PMC3283691; doi:10.1371/journal.pone.0031852)
Supplement: Text S4 — Details of SAXS Experiments. (DOCX) [file pone.0031852.s009.docx]

***SUPPORTING INFORMATION***

**Text S4:**

***SAXS Experiments* –** SAXS data for LBD and DBD-LBD constructions of PPAR monomer and PPAR/RXR heterodimer were obtained through measurements of proteins at different concentrations (1, 3 and 6 mg/mL). All the x-ray scattering curves obtained in these different conditions were practically identical, indicating the absence of spatial correlation effects over the applied concentration range. This evidence can also be verified through *R_g_* values calculated for every concentrations tested (Table S1). The variation between the *R_g_* values for different concentrations was insignificant, showing that their values are independent of protein concentration for all the samples analyzed.
