# Supplementary material for: Low-Resolution Molecular Models Reveal the Oligomeric State of the PPAR and the Conformational Organization of Its Domains in Solution
Source: PLoS One. 2012 Feb 21;7(2):e31852. doi: 10.1371/journal.pone.0031852 (PMC3283691; doi:10.1371/journal.pone.0031852)
Supplement: Text S5 — Absolute scale SAXS measurements. (DOCX) [file pone.0031852.s010.docx]

***SUPPORTING INFORMATION***

**Text S5:**

***Absolute scale SAXS measurements* -** The absolute intensity scale for the SAXS data can be used to compare the experimental scattering curves between different samples. In particular, this approach was used to determine the molecular weight of proteins to analyze their oligomeric form.

In order to calculate the absolute intensity, the scattering from an empty cell, water and buffers were measured first. All measurements were carried out at 20ºC. To calculate the forward scattering I(0) in the absolute scale, the known scattering of water of 1.632x10^-2^ cm^-1^ at 288 K was used [1]. By dividing the relative I(0)_s_ of the proteins with the experimental constant scattering of water and then multiplying by the absolute scattering of water one obtains the I(0)_s_ of the proteins in absolute scale. To calculate the molecular weight (MW) in kDa we used the following formula [1,2].

$$MW=\frac{I(0)}{c}\frac{N_{a}}{{\Delta p}_{m}^{2}}$$

where I(0)/c is the forward scattering normalized against concentration, ${\Delta p}_{m}$= 2.08 10^10^ cm/g is the scattering contrast per mass and *N_a_* = 6.023x10^23^ mol^-1^ is the Avogadro number [3].
