# Supplementary material for: Low-Resolution Molecular Models Reveal the Oligomeric State of the PPAR and the Conformational Organization of Its Domains in Solution
Source: PLoS One. 2012 Feb 21;7(2):e31852. doi: 10.1371/journal.pone.0031852 (PMC3283691; doi:10.1371/journal.pone.0031852)
Supplement: Text S6 — The proteins molecular weight determination by SAXS measurements using BSA as a reference. (DOCX) [file pone.0031852.s011.docx]

***SUPPORTING INFORMATION***

**Text S6:**

***The proteins molecular weight determination by SAXS measurements using BSA as a reference* –** Bovine serum albumin (BSA) was purchased as a lyophilized powder from Sigma-Aldrich. A solution with BSA concentration of 1 mg/mL was prepared by directly dissolving the corresponding amount of protein powder in the same samples buffer, and BSA concentration were determined by UV absorption on 280 nm.

Through the measures of samples among known proteins concentrations and a proper BSA standard, the molecular weights of the samples can be estimated analyzing the initial intensity at zero angle I(0) [1].
