# Supplementary material for: Low-Resolution Molecular Models Reveal the Oligomeric State of the PPAR and the Conformational Organization of Its Domains in Solution
Source: PLoS One. 2012 Feb 21;7(2):e31852. doi: 10.1371/journal.pone.0031852 (PMC3283691; doi:10.1371/journal.pone.0031852)
Supplement: Text S7 — Hydrogen-deuterium exchange experiments analyzed by mass-spectrometry. (DOCX) [file pone.0031852.s012.docx]

***SUPPORTING INFORMATION***

**Text S7:**

***Hydrogen-deuterium exchange experiments analyzed by mass-spectrometry* –** These experiments were conducted in order to analyze the dynamics of protein in solution and to evaluate the protein surfaces involved in the complex formation. The differences in deuterium incorporation between the preparations reflect increased compactness and lower flexibility of the more structured complexes with cognate DNA and/or heterodimerization partner, in comparison to the hPPARγ alone. As a result, the H/D-Ex of hPPARγ/hRXRα complex, we identified 51 peptides for hPPARγ, covering 92% of its amino acid sequence (Figure S4 A). In addition, through measures of different deuterium incubation times, the kinetics of deuterium incorporation is fast and 15 and 30 minutes of incubation experienced no expressive variation (Figure S4 B).
